# Energy Density, Portion Size, and Eating Occasions: Contributions to Increased Energy Intake in the United States, 1977–2006

**DOI:** 10.1371/journal.pmed.1001050

**Published:** 2011-06-28

**Authors:** Kiyah J. Duffey, Barry M. Popkin

**Affiliations:** 1 Department of Nutrition, Gillings School of Global Public Health, University of North Carolina at Chapel Hill, Chapel Hill, North Carolina, United States of America; 2 Carolina Population Center, University of North Carolina at Chapel Hill, Chapel Hill, North Carolina, United States of America; Harvard University, United States of America

## Abstract

**Background:**

Competing theories attempt to explain changes in total energy (TE) intake; however, a rigorous, comprehensive examination of these explanations has not been undertaken. Our objective was to examine the relative contribution of energy density (ED), portion size (PS), and the number of eating/drinking occasions (EOs) to changes in daily TE.

**Methods and Findings:**

Using cross-sectional nationally representative data from the Nationwide Food Consumption Survey (1977–78), Continuing Survey of Food Intakes of Individuals (1989–91), and National Health and Nutrition Examination Surveys (1994–98 and 2003–06) for adults (aged ≥19 y), we mathematically decompose TE (kcal/d) to understand the relative contributions of each component—PS (grams/EO), ED (kcal/g/EO) and EO(number)—to changes in TE over time. There was an increase in TE intake (+570 kcal/d) and the number of daily EOs (+1.1) between 1977–78 and 2003–06. The average PS increased between 1977–78 and 1994–98, then dropped slightly between 1994–98 and 2003–06, while the average ED remained steady between 1977–78 and 1989–91, then declined slightly between 1989–91 and 1994–98. Estimates from the decomposition statistical models suggest that between 1977–78 and 1989–91, annualized changes in PS contributed nearly 15 kcal/d/y to increases in TE, while changes in EO accounted for just 4 kcal/d/y. Between 1994–98 and 2003–06 changes in EO accounted for 39 kcal/d/y of increase and changes in PS accounted for 1 kcal/d/y of decline in the annualized change in TE.

**Conclusions:**

While all three components have contributed to some extent to 30-y changes in TE, changes in EO and PS have accounted for most of the change. These findings suggest a new focus for efforts to reduce energy imbalances in US adults.

*Please see later in the article for the Editors' Summary*

## Abstract 2

Using data from three surveys, Kiyah Duffey and Barry Popkin found that changes in eating/drinking occasions and portion size consistently account for most of the change in daily total energy intake over a 30-year period.

## Editors' Summary

**Background:**

Since the mid 1970s, the proportion of people who are obese (people who have an unhealthy proportion of body fat) has increased sharply in many countries. In the US, the proportion has doubled since 1980, and a third of all US adults—more than 72 million people—are now classified as obese. That is, they have a body mass index (BMI, an indicator of body fat calculated by dividing a person's weight in kilograms by their height in meters squared) of greater than 30. Compared to people with a healthy weight (a BMI between 18.5 and 25), obese individuals and overweight people (who have a BMI between 25 and 29.9) have an increased risk of developing diabetes, heart disease, and stroke and tend to die younger. People become unhealthily fat by consuming food and drink that contains more energy (kilocalories, or kcal) than they need for their daily activities. In these circumstances, the body converts the excess energy into fat stores.

**Why Was This Study Done?:**

Because obesity causes illness and premature death, it is essential that the obesity epidemic is halted and, if possible, reversed. But before public health policies can be formulated to prevent obesity, we need to understand what is driving the epidemic. Many experts believe that increases in the total daily intake of energy from food and drink, irrespective of changes in physical activity, are enough to explain the observed increases in weight at the population level since the 1970s. But why has total energy intake increased? Three main causes have been proposed—an increase in the frequency of meals and snacks (eating occasions), increases in the typical food and drink portion sizes, and changes in the energy density of the foods and drinks consumed. In this study, the researchers use data from US food surveys to examine the relative contributions made by these three variables to changes in daily total energy intake between 1977 and 2006.

**What Did the Researchers Do and Find?:**

The researchers used a technique called “mathematical decomposition” to analyze cross-sectional, nationally representative dietary intake data for US adults collected in food surveys undertaken in 1977–78, 1989–91, 1994–98, and 2003–06. Cross-sectional surveys examine a group of people at a single time point; food surveys collect information about all the food and drink consumed by individuals over a 24-hour period. The average daily total energy intake increased from 1,803 kcal in 1977–78 to 2,374 kcal in 2003–06, an increase of 570 kcal. In the last decade of the study alone, the average daily energy intake increased by 229 kcal. Between 1977–78 and 1989–91, changes in portion size accounted for an annual increase in the daily total energy intake of nearly 15 kcal, whereas changes in the number of eating occasions accounted for an increase of just 4 kcal. By contrast, between 1994–98 and 2003–06, changes in the number of eating occasions accounted for an annual increase in daily total energy intake of 39 kcal, whereas changes in portion size accounted for an annual decrease in daily energy intake of 1 kcal. Changes in the energy density of food and drink accounted for a slight decrease in daily total energy intake over the 30-year study period.

**What Do These Findings Mean?:**

These findings indicate that, although the energy density of food and drink, portion size, and the number of meals and snacks per day have all contributed to changes in the average daily total energy intake of US adults over the past 30 years, increases in the number of eating occasions and in portion size have accounted for most of the change. The accuracy of these findings may be affected by the use of self-reporting in the food surveys (people tend to underestimate the calorie content of “junk” food) and by the mathematical formula used to assess the relative contribution of each component of daily energy intake. Nevertheless, these findings suggest that efforts to prevent obesity among US adults (and among adults in other developed countries) should focus on reducing the number of meals and snacks people consume during the day as a way to reduce the energy imbalance caused by recent increases in energy intake.

**Additional Information:**

Please access these Web sites via the online version of this summary at http://dx.doi.org/10.1371/journal.pmed.1001050.

- The US Centers for Disease Control and Prevention provides information on all aspects of overweight and obesity (including some information in Spanish)
- The UK National Health Service Choices Web site also provides detailed information about obesity
- The International Obesity Taskforce provides information about the global obesity epidemic
- The US Department of Agriculture's MyPyramid.gov Web site provides a personal healthy eating plan; the Weight-control Information Network is an information service provided for the general public and health professionals by the US National Institute of Diabetes and Digestive and Kidney Diseases (in English and Spanish)
- MedlinePlus has links to further information about obesity (in English and Spanish)

## Introduction

In the context of the growing obesity epidemic [1], it has been suggested that increases in caloric availability and thus energy intake [2], irrespective of changes in physical activity, are enough to explain the observed increases in weight at the population level [3]. Theories about the causes of change in energy intake are numerous, but tend to focus on one of three areas: increases in the frequency of eating/drinking occasions (EOs) [4]–[6], especially snacking [7]; increases in the typical portion sizes (PSs) of foods and beverages [8]–[11]; or changes in the energy density (ED) of the foods consumed (termed “volumetrics” by Rolls and colleagues) [12]–[14].

Much of this research focuses on the effect of the ED or PSs of individual foods, or preload conditions [15],[16], on energy intake at a given meal The limited epidemiological work has been cross-sectional [17]. At least one study [18] also examined whether increasing PS has an effect beyond a single EO, reporting that increased PSs over 2 d resulted in increased energy intake and that the increase on day one was not compensated for on the second day [18]. Several studies have also confirmed that when individuals consume meals that are lower in ED, their daily energy intake is also lower [19]–[22]. Taken together these findings suggest that it is the total meal (combination of foods and beverages consumed at a given EO), not just individual foods consumed, that is important in determining total energy (TE) intake and should be the focus of research.

A small body of research has examined the combined effect of changes in both the ED and PS of foods with respect to energy intake [23]–[25], but to our knowledge similar research does not exist for the other possible combinations of ED, EO frequency, and PS, nor have these factors been examined all together in either large-scale epidemiological studies or clinical trials. Further, the research on PSs has focused mainly on separate foods and beverages (e.g., sugar-sweetened beverages or cheeseburgers), ignoring both their potential effect on each meal or snack occasion and the relationship of overall PSs of all other meals and snacks to daily totals.

To address this knowledge gap, in the present study we examine the relative contribution of changes in the frequency of EOs, PS for each EO, and ED for each EO to changes in TE intake using nationally representative samples of US adults between 1977 and 2006.

## Methods

### Study Population

Cross-sectional nationally representative dietary intake data of adults 19 y and older were taken from four US food surveys. United States Department of Agriculture (USDA) data came from respondents of the Nationwide Food Consumption Survey (NFCS) of 1977–78 (*n* = 17,464) and the Continuing Survey of Food Intakes of Individuals (CSFII) 1989–91 (*n* = 8,340) and 1994–96, 1998 (CSFII 1994–98, *n* = 9,460). We also combined two consecutive National Health and Nutrition Examination Survey (NHANES) surveys, 2003–04 and 2005–06, into a single analytic sample (NHANES 2003–06, *n* = 9,490). The USDA and NHANES surveys are based on stratified area probability samples of non-institutionalized US households in the 48 contiguous [26] or all 50 states [27]. Detailed information about each survey and its sampling design has been previously published [26]–[29], and a comparison of the sampling and 24-h recall intake methodologies can be found in Table S1. The study was approved by the Institutional Review Board at the University of North Carolina at Chapel Hill.

### Dietary Data

The NFCS 1977–78 and CSFII 1989–91 surveys collected dietary intake data over three consecutive days using a single-interviewer-administered multiple-pass 24-h dietary recall followed by a self-administered 2-d diet record using methods developed by the USDA. Dietary data from these surveys consisted of all foods consumed at and away from home (24-h recall) and a comprehensive record of all foods eaten on the day of the interview and the following day (2-d record). This USDA dietary methodology was later integrated into the CSFII 1994–98 and NHANES 2003–06 surveys, which utilized two nonconsecutive days of interviewer-administered 24-h dietary recalls (3–10 d apart). In order to maintain consistency across studies we utilized the first day of available 24-h recall dietary data. We excluded all reported instances of water being consumed as a separate food item from all surveys, as this information was not collected in the same manner across exams. In the later NHANES exams, water (as a beverage) was probed for specifically, which resulted in a dramatic increase in the reported instances of water consumption.

### Food and Beverage Definitions

Foods and beverages were defined and grouped according the UNC-CH food-grouping system [30]. Briefly, foods and beverages were grouped into 101 nutrient-based food groups (including 16 beverage groups) according to fat and fiber content. Because “dish” identifiers are not available in NHANES, it is not possible to accurately and confidently link foods consumed separately but which might constitute a single dish, e.g., milk and cereal consumed at the same EO. In instances such as this, cereal is identified as a food, while milk is identified as a beverage. Although it seems possible to make educated guesses about foods like milk and cereal, there are many more assumptions required to assign a single “food” status to something like milk consumed with macaroni and cheese. Therefore, in all cases where any beverage was consumed in the same EO as a food, the beverage is considered independent of the food. The UNC-CH food-grouping system has been used previously in studies of beverage [2],[31] and dietary intake [32],[33] specifically, as well as studies examining snacking [7],[34] and overall eating behaviors [35],[36].

### Defining Eating Occasions

EOs, either meals or snacks, were self-defined by respondents in both the USDA and NHANES surveys. Meals were defined by the respondent as breakfast/brunch, lunch, and dinner/supper, while the snack category included those EOs defined by the respondent as “snack,” plus related snacking occasions (i.e., food and/or coffee/beverage breaks). All occasions that were identified as snacks but were consumed within 15 min of each other were combined into a single snacking event. Also, some people defined foods eaten at the same time as both a snack and a meal. As an example, suppose an individual reported consuming a sandwich and a bag of chips (eaten at the same time). This individual identified the sandwich as lunch and the chips as a snack. In instances where this occurred, both items were considered eaten as part of a single EO (lunch), rather than as two separate EOs (lunch and a snack).

Beverages consumed alone, and not identified as a meal, were considered snacks. The number of meals and snacks was then summed for each individual for a total number of EOs. All foods were assigned to a specific EO in 1977–78 and in 2003–06, while in the other two surveys 0.15% of food items were not assigned because they had neither an EO name nor a time associated with the EO. This method of assigning meals and snacks has been previously employed to study overall eating [37] and snacking behavior [7] in a sample of US adults.

### Total Energy, Portion Size, and Energy Density

We calculated per EO measures for energy intake, PS, and ED. For each individual, the daily total gram weight (PS) and total daily energy of all foods consumed were summed over a 24-h period and divided by the total number of EOs as a measure of per EO PS and per EO energy. ED was then generated by dividing energy (per EO) by PS (per EO). This was done for foods and beverages separately.

### Decomposition Algorithm

Mathematical decomposition has been applied to many measures of changes in health and behavior (e.g., mortality and fertility rates [38]–[40]). We define total daily energy intake (TE) (kcal/d) as the number of daily EOs multiplied by the average PS (grams) per EO multiplied by the average ED (kcal/g) of each EO, as in the following equation:
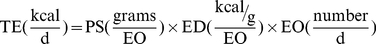
(1)


Using this equation, we then estimate the proportionate contribution (a partial derivative) of changes in each of these components to overall changes in total daily energy intake by taking the derivative of changes in TE with respect to changes in PS, ED, and the number of EOs, holding the other two factors constant at their mean [38]. Briefly, for each component of total daily energy, the change between two time points (e.g., 1977–78 and 1989–91) is multiplied by the average of the other two components. This derivative is calculated for each component (PS, ED, and EOs) and the values summed to generate the full derivative for change in TE intake, as shown in the following equation:
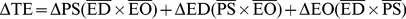
(2)


To annualize change, these values were then divided by the number of years between each wave of data collection (i.e., results comparing 1977–78 to 1989–91 were divided by 12.5 [mean year points: 1990–1977.5 = 12.5]). The resulting output is interpreted as the annual change in energy (kcal/d/y) that is attributed to changes in PS, ED, and EOs, with sign indicating the direction of change.

### Statistical Analysis

All analyses were conducted using Stata 11 (StataCorp). We used survey commands to account for survey design: weighting and clustering. All values were adjusted to the 1977–78 age–gender–race/ethnicity sample distribution and are reported as mean (or percent) and standard error. Values were then annualized to account for the unequal spacing within and between exam years. To test for statistical differences in sample characteristics (not PS, ED, EO, or TE) comparing all years to each other, we used independent two-sided *t* tests with *p*≤0.05 set for statistical significance using the Bonferroni correction for multiple comparisons.

## Results

### Overall

The sample population in 1977–78 was significantly younger and had a higher percentage of non-Hispanic white males with 12 or fewer years of education compared to the later exam years. The population in 1977–78 also had a lower percentage of individuals of Hispanic and “non-Hispanic other” race/ethnicity and a lower percentage of persons living at or above the 350% poverty income ratio (Table 1).

**Table 1:** Characteristics of study populations across exam years.

Sample Characteristic	Subcategory	Exam Periods			
		1977–78	1989–91	1994–98	2003–06
**Sample size**		17,228	10,501	9,338	9,018
**Age (y)**		44±0.26	45±0.46	45±0.36	46±0.48^a^
**Female (%)**		41±0.52	53±0.66^a^	52±0.63^a^	52±0.44^a^
**Race/ethnicity (%)**	Non-Hispanic white	83±1.33	80±1.16	76±1.84^a^	72±2.15^a^^,^ b
	Non-Hispanic black	11±1.06	11±0.62	11±1.05	11±1.31
	Hispanic	5±0.66	7±1.07	9±1.48^a^	11±1.34^a^^,^ b
	Non-Hispanic other	1±0.15	2±0.59^a^	4±0.44^a^^,^ b	5±0.43^a^^,^ b
**Education (%)**	<12 y	32±1.06	19±0.68^a^	16±0.82^a^^,^ b	17±0.94^a^
	12 y/GED	32±0.68	35±0.58^a^	35±1.07^a^	26±0.72^a^^,^ b ^,^ c
	13–15 y (<BA)	17±0.56	22±0.36^a^	24±0.56^a^^,^ b	32±0.77^a^^,^ b ^,^ c
	16+ (BA+)	19±0.79	23±0.66^a^	26±1.43^a^	25±1.40^a^
**Poverty income ratio**	<180%	28±0.95	24±0.74^a^	26±1.13	28±1.32^b^
	180% to <350%	38±0.60	30±1.14^a^	31±0.78^a^	28±1.01^a^^,^ c
	≥350%	35±1.02	46±1.85^a^	43±1.43^a^	43±1.59^a^
**Components of TE** d	PS (g/EO)	523±3.2	573±4.3	590±6.7	588±7.6
	ED (kcal/g/EO)	0.97±0.004	0.97±0.005	0.95±0.006	0.95±0.007
	EOs (number)	3.8±0.03	3.9±0.04	4.3±0.04	4.9±0.04
	Total daily energy (kcal)^d^	1,803±12.6	1,949±13.4	2,145±25.1	2,374±17.8

Values are mean ± standard error. Data are from cross-sectional nationally representative samples of adults (≥19 y) taken from NFCS 1977–78, CSFII 1989–91 and 1994–98, and NHANES 2003–06.

a Values are different from 1977–78, *p*<0.05 using Bonferroni-corrected two-sided Student's *t* test.

b Values are different from 1989–91, *p*<0.05 using Bonferroni-corrected two-sided Student's *t* test.

c Values are different from 1994–96, *p*<0.05 using Bonferroni-corrected two-sided Student's *t* test.

d Values are standardized to the age, race, and gender distributions of 1977–78 sample population using predicted means. Predicting violates the assumption of independence required for performing Student's *t* tests of means; therefore, statistically significant differences are not calculated for these measures.

BA, Bachelor of Arts; GED, General Equivalency Diploma.

The average PS per EO increased from 1977–78 to 1989–91 (+49 g) and again from 1989–91 to 1994–98 (+18 g), before declining slightly in 2003–06 (−2 g). The average ED per EO did not change between 1977–78 and 1989–91, then declined slightly between 1989–91 and 1994–98 (−0.02 kcal/g/EO). The total number of daily EOs increased between each exam period, from 3.8 EO/d in 1977–78 to 4.9 EO/d in 2003–06 (Table 1).

Total daily energy intake increased by 570 kcal/d between 1977–78 and 2003–06 (Table 1). Between each exam period, there were increasingly greater differences in predicted TE intake, with the largest increase occurring in the last decade (1994–98 to 2003–06, +229 kcal/d).

### By Food and Beverage

**Figure 1:**
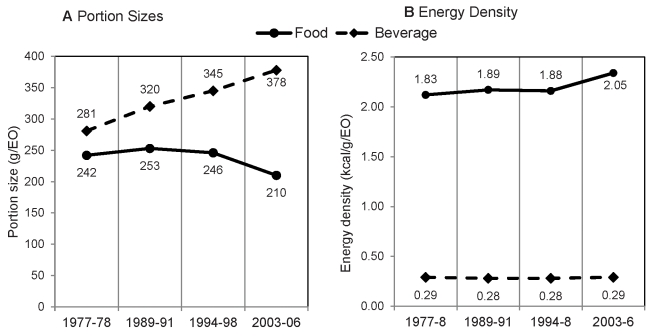
Average portion size and energy density per eating occasion, by food and beverage. Data on PS (A) and ED (B) are from cross-sectional nationally representative samples of adults (≥19 y) taken from NFCS 1977–78 (*n* = 17,464), CSFII 1989–91 (*n* = 8,340) and 1994–98 (*n* = 9,460), and NHANES 2003–06 (*n* = 9,490), standardized to the age, gender, and race/ethnic distribution of the sample in 1977–78.

The average PS per EO of beverages increased (1977–78 to 2003–06, +97 g) between each exam period, whereas the average PS per EO of foods increased between 1977–78 and 1998–91 (+11 g), then declined to its lowest value in 2003–06 (Figure 1A). ED, on the other hand, remained relatively stable between exam years (Figure 1B). This is especially true for beverages, which showed virtually no change in ED per EO between 1977–78 and 2003–06. The ED of foods showed more fluctuation, hovering between 1.83 and 1.89 kcal/g/EO until 2003–06, when the average ED per EO increased to 2.05 kcal/g/EO (Figure 1B). It is important to keep in mind, however, that although there were larger changes in the PS of beverages, changes in the PS of foods still had a greater impact on energy intake. For example, between 1977–78 and 2003–06, changes in food energy provided an additional 367 kcal/d while changes in beverage energy provided only an additional 203 kcal/d (data not shown).

### Decomposing Change in Total Energy

**Figure 2:**
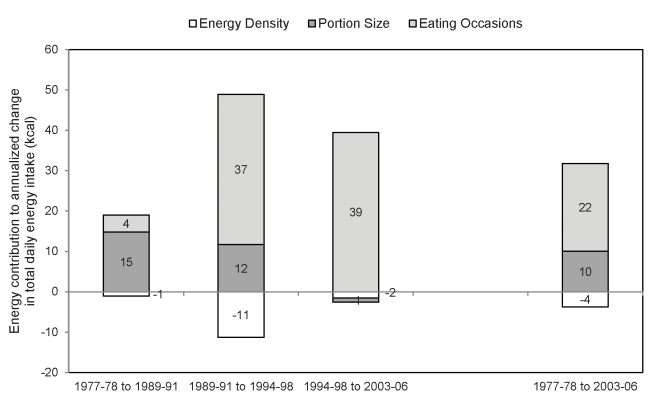
Annualized contribution of portion size, energy density, and eating occasions to total energy intake changes. Values represent the annualized energy contribution (kcal) of changes in the number of EOs, PS, or ED of each EO to changes in total daily energy intake (kcal). Data are from cross-sectional nationally representative samples of adults (≥19 y) taken from NFCS 1977–78 (*n* = 17,464), CSFII 1989–91 (*n* = 8,340) and 1994–98 (*n* = 9,460), and NHANES 2003–06 (*n* = 9,490), standardized to the age, gender, and race/ethnic distribution of the sample in 1977–78.

Over the 30-y period, all three components (ED, PS, and EO) contributed to a greater or lesser extent to changes in TE intake (Figure 2). For example, between 1977–78 and 1989–91, increases in the number of EOs accounted for just 4 kcal/d/y of the annualized increase in TE intake; this jumped to 37 kcal/d/y between 1989–91 and 1994–98 and 39 kcal/d/y between 1994–98 and 2003–06 (Figure 2). Increases in PS accounted for the largest energy change between 1977–78 and 1989–91 (15 kcal/d/y), but PS's contribution to annualized change in TE dropped between 1989–91 and 1994–98 (Figure 2). Between 1994–98 and 2003–06, PS accounted for −1 kcal/d/y of the change in TE, which means that decreases in PS (and ED) offset increases in the number of EO over this time period. Over time, changes in ED have partially offset changes in the other two variables, accounting for −1 kcal/d/y of the observed change in TE intake between 1977–78 and 1989–91 and −11 kcal/d/y of the observed change in TE between 1989–91 and 1994–98. Looking at changes over the full 30-y period, the largest contributor to change in TE intake was change in the frequency of EO, accounting for 22 kcal/d/y of the observed annualized change in TE.

## Discussion

To our knowledge this is the first study to examine the combined contribution of changes in PS, ED, and the frequency of EOs to changes in total daily energy intake in any free-living population. Annualized daily energy intake for US adults increased by 28 kcal/d/y between 1977–78 and 2003–06. Among adults in the US, we show that for the full period between 1977 and 2006 the largest contributor to change in annualized total daily energy intake was change in the number of EOs, accounting for roughly 22 kcal/d/y. Over the full time period, changes in PS accounted for the next largest proportion, at 10 kcal/d/y, and our results suggest that the ED of the average EO has actually decreased, offsetting increases in PS and the number of EOs and accounting for roughly −4 kcal/d/y of the annualized change in TE. It is important to note that this is a macro level analysis; these results do not negate the issue of how individual diet and weight change are affected by ED, PS, or eating frequency. However, to the extent that all energy intake is equal, and has an equal impact on energy imbalance, this approach to studying changes in energy intake helps guide us to interventions to reduce intake.

Our findings are in line with more detailed EO-specific research from our group, which documents increases in the frequency of reported EOs among US adults [37]. At the 50^th^ percentile, the average number of EOs increased from 3.5/d to 5.0/d, a change that was accompanied by an increase of 400 kcal/d from meals and snacks combined [37].

The supersizing of portions of various food and beverage items has been the subject of much scientific research and both scientific and popular books and films [41]–[45]. Our group has shown actual consumption of key food and beverage portions increased across all EOs [46]. Other research shows similar increases for selected foods in the last decade, although much of this research has focused on specific food items [42],[47] (i.e., soft drinks, hamburgers, and pizza) as opposed to changes in total meal size. Rolls et al. [23] have also studied combinations of water and food at meals. They report that decreasing the ED (and increasing the volume) of a meal preload by adding water results in a reduction of energy intake at lunch; giving the equivalent amount of water as a beverage separate from a meal did not affect satiety and was associated with greater energy intake compared to the condition in which water was incorporated into the meal [23].

The present study employs just one possible method of decomposition; utilizing the fact that daily energy intake can be defined as the number of EOs (number) multiplied by PS (grams) and ED (kcal/g) to calculate the partial and full derivatives of each of these components as contributors to overall change. Decomposing change has been previously employed, especially in the sociological literature (i.e., examining changes in fertility rates), and other methods exist [39],[40],[48],[49] and should be examined. In addition, the use of nationally representative cross-sectional dietary data has its limitations, particularly for trend analyses. Perhaps the most notable limitation is the introduction of the five-step multiple-pass method of 24-h recall collection, which was implemented in the NHANES 2003–04 (and subsequent 2005–06) survey. This differs from previous USDA methodologies, and could result in more accurate intake reported by individuals in later years since there is additional prompting by the interviewer. Since there are no bridging studies to determine the extent to which these methodological shifts may have resulted in systematic changes in individuals' reporting, it is not possible to know whether such confounding by time exists. Bridging studies conducted as a result of methodological shifts between the 1970s and 1980s, however, found that shifts in TE and food composition did not significantly impact results [50],[51].

Additionally, in NHANES 2003–04 and 2005–06, respondents were specifically asked if they consumed water (e.g., it was added as a food item). This probing resulted in the reporting of 26,000 “water only” EOs in 2003–06 (approximately 24% of reported beverage consumption; data not shown), an increase compared to previous years, which had 200–300 observations each (range 0.06%–0.02% of reported beverage consumption; data not shown). This survey change had significant implications for the calculation of ED and the number of EOs specifically, and inclusion dramatically alters our decomposition results between 1994–98 and 2003–06, inflating the number of reported EOs and decreasing the ED of the average EO. Since this additional probing was not consistent between exams or across exam years, we excluded these additional water-only EOs to maintain consistency across time.

Another important limitation with using cross-sectional data is the possibility that results are confounded by population-level changes in factors that might influence our relationship of interest. Although we can at least partially control for some of these changes (through age, gender, and race/ethnic standardization to the earliest time point), we are unable to adjust for others (such as physical activity or the prevalence of chronic disease) because of a lack of detailed and comparable measures over time. Finally, we were limited by the possibility of increasing underestimates of actual food intake over time. Scholars have shown that adults tend to underestimate TE intake, particularly from “junk foods” and other foods that are considered to have negative health connotations [52]–[55]. This is particularly true for overweight individuals [56],[57].

Using cross-sectional nationally representative samples of US adults, this study documents marked increases in the number and PSs of EOs and steady overall ED per EO over the past 30 y. During the most recent period, from 1994–98 to 2003–06, there were large increases in the number of EOs, but, equally important, there were no changes in the average ED of each EO. The results suggest that as contributors to increased caloric intake over both this most recent decade and over the full 30-y period, increased EO contributed significantly more to the shift in TE intake than the other two components, although PS positively and ED negatively contributed to some extent. To the extent that energy imbalance as a result of increased energy intake contributes to obesity and its associated co-morbidities, prevention efforts should focus more on reducing EOs as a way to reduce energy imbalance.

## Supporting Information

Table S1 **Description of sampling schemes and dietary assessment methods across survey years.**
(DOC)

## Abbreviations

CSFII: Continuing Survey of Food Intakes of Individuals
ED: energy density
EO: eating/drinking occasions
NFCS: Nationwide Food Consumption Survey
NHANES: National Health and Nutrition Examination Survey
PS: portion size
TE: total energy
USDA: United States Department of Agriculture
