# Supplementary material for: Energy Density, Portion Size, and Eating Occasions: Contributions to Increased Energy Intake in the United States, 1977–2006
Source: PLoS Med. 2011 Jun 28;8(6):e1001050. doi: 10.1371/journal.pmed.1001050 (PMC3125292; doi:10.1371/journal.pmed.1001050)
Supplement: Table S1 — Description of sampling schemes and dietary assessment methods across survey years. (DOC) [file pmed.1001050.s001.doc]

Table S1: Description of sampling schemes and dietary assessment methods across survey years.

| **Survey** | **Years** | **Sampling framework** | **Sample weights used** | **24-hour recall assessment method** |
| --- | --- | --- | --- | --- |
| Nationwide Food Consumption Survey (NFCS) | 1977-78 | - Stratified, multistage, national probability sample of noninstitutionalized civilian population in 48 contiguous states. | Yes | - 1 (one) in-person interviewer administered 24-hour recall. |
| Continuing Survey of Food Intake in Individuals (CSFII) | 1989-91 | - Stratified, multistage, national probability sample of noninstitutionalized civilian population in 48 contiguous states - Oversampling of low-income populations. | Yes | - 1 (one) in-person interviewer administered 24-hour recall. |
|  | 1994-98 | - Stratified, multistage, national probability sample of noninstitutionalized civilian population in all 50 states (1994-96, 1998). - Oversampling of young children, low-income population conducted in 1998. | Yes | - 2 (two) non-consecutive interviewer administered in-person 24-hour recall using the automated 3-step multiple pass method. |
| National Health and Nutrition Examination Survey (NHANES) | 2003-06 | - Stratified, multistage, national probability sample of noninstitutionalized civilian population. - Oversampling of African Americans, Mexican Americans, low-income White Americans, adolescents (aged 12-19 y), older adults (aged 60+ y). | Yes | - 2 (two) non-consecutive computer-assisted interviewer administered 24-hour recall using the automated 5-step multiple pass method. |
